# Hin-mediated DNA knotting and recombining promote replicon dysfunction and mutation

**DOI:** 10.1186/1471-2199-8-44

**Published:** 2007-05-25

**Authors:** Richard W Deibler, Jennifer K Mann, De Witt L Sumners, Lynn Zechiedrich

**Affiliations:** 1 Interdepartmental Program in Cell and Molecular Biology, Baylor College of Medicine, Houston, Texas 77030-3411 USA; 2 Department of Molecular Virology and Microbiology, Baylor College of Medicine, Houston, Texas 77030-3411 USA; 3 Department of Systems Biology, Harvard Medical School, Boston, Massachusetts 02115 USA; 4 Department of Mathematics, Florida State University, Tallahassee, Florida 32306-4510 USA

## Abstract

**Background:**

The genetic code imposes a dilemma for cells. The DNA must be long enough to encode for the complexity of an organism, yet thin and flexible enough to fit within the cell. The combination of these properties greatly favors DNA collisions, which can knot and drive recombination of the DNA. Despite the well-accepted propensity of cellular DNA to collide and react with itself, it has not been established what the physiological consequences are.

**Results:**

Here we analyze the effects of recombined and knotted plasmids in *E. coli *using the Hin site-specific recombination system. We show that Hin-mediated DNA knotting and recombination (i) promote replicon loss by blocking DNA replication; (ii) block gene transcription; and (iii) cause genetic rearrangements at a rate three to four orders of magnitude higher than the rate for an unknotted, unrecombined plasmid.

**Conclusion:**

These results show that DNA reactivity leading to recombined and knotted DNA is potentially toxic and may help drive genetic evolution.

## Background

Much of DNA metabolism is understood in the context of the linear sequence of nucleotides that compose the nucleic acid. For example, gene promoters, replication origins, partitioning sequences and genes themselves are defined by their particular DNA sequences. However, the physical, mechanical and topological properties of DNA also exert significant influence over DNA metabolism [1]. Inside cells, the long (1.6 mm for *Escherichia coli*) and flexible (persistence length ≈ 50 nm) DNA must be compacted into a very small volume, achieving a liquid crystalline state of 80 – 100 mg/ml [2-4]. Understanding how DNA functions requires understanding its conformation under such compact conditions.

DNA conformation is affected not only by crowding but also by its physical structure. Intuitively, anything long, thin and flexible can become self-entangled. Interestingly, for 200 kb DNA molecules at thermal equilibrium, the most energetically favorable conformation is the trefoil knot, 3_1 _[5]. 200 kb is ~20-fold smaller than the chromosome of *E. coli*. Thus, it is not surprising that when cells are lysed, a small portion (~1%) of plasmid DNA, which is only on the order of 4 kb, is found knotted [6-10]. The propensity for DNA to knot is predicted to be even greater for the longer and more folded eukaryotic chromosomes [11]. However, if we apply the figure of 1% DNA knotting to human chromosomes, then nearly every other diploid human cell would have a knot.

**Figure 1:**
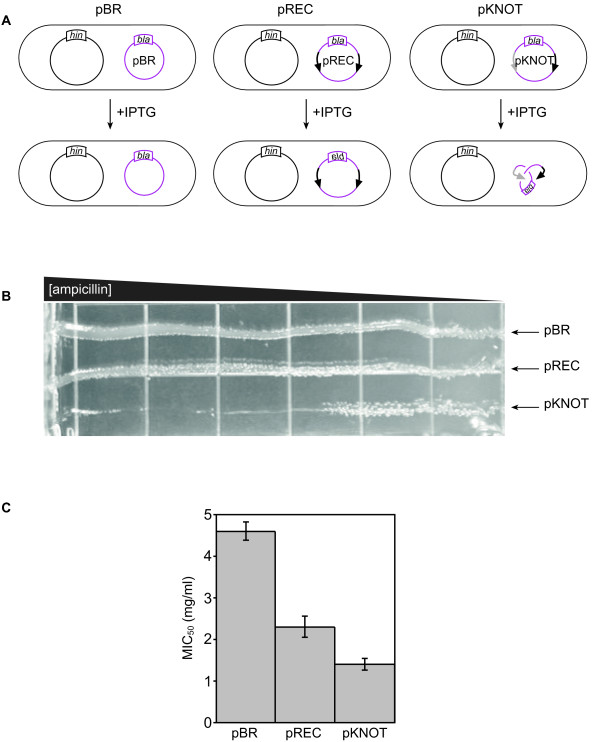
**Physiological effects of Hin-mediated recombination/knotting**. (A) Assay for the effect of knotting on the function of a gene. The ovals represent *E. coli *cells. The Hin expression vector, pHIN, and plasmid substrates pBR, pREC and pKNOT containing the *bla *gene (encoding β-lactamase) are depicted. Wild-type recombination sites are depicted as black arrows. The mutant *hix *site is shown as a grey arrow. (B) Effect of DNA knotting on ampicillin sensitivity of *E. coli *strain W3110 containing pHIN and either pBR, pREC or pKNOT. Single colonies were streaked from left to right across LB-agar that contained an ampicillin gradient and constant IPTG (1 mM) and spectinomycin (50 μg/ml) for Hin overexpression and maintenance. The experiment was repeated five times in either strain C600 or W3110, and was carried out either from high to low or from low to high ampicillin concentration with identical results. (C) Ampicillin sensitivity (MIC_50_) was quantified using the plate dilution method.

Although DNA knotting is clearly energetically favorable for DNA, several observations suggest that the intracellular environment should further exacerbate knotting. Experiments with the bacteriophage P4 demonstrated that the confinement of DNA in a small volume stimulates the knotting of DNA [12]. Furthermore, DNA inside the cell is negatively supercoiled. Negative supercoiling promotes a number of genetic processes, including gene expression and DNA replication, in part because it promotes opening of the DNA duplex [13-16]. DNA supercoiling also compacts the DNA and brings distant strands into close proximity [17,18]. As a consequence, supercoiling promotes strand collision and DNA tangling. Indeed, computer simulations have revealed that supercoiling should drive DNA knotting because writhe in a knot is less stressful on the DNA than writhe in an unknotted, supercoiled molecule [19,20].

Collisions of DNA helices with one another are potentially problematic because DNA is a self-reactive molecule. The repair of double strand breaks, single strand gaps and stalled replication forks involve recombination, which requires physical contact with a homologous DNA molecule. Similarly, transposition, site-specific recombination and modulation of transcription (by enhancers and other *cis*-regulatory elements) often involve DNA-DNA interactions. However, it has not been well established whether DNA strand collisions and the potential resulting entanglements affect DNA metabolism in the cell.

One indication that DNA knotting is deleterious to cells is the universal prevalence of type-2 topoisomerases. These are essential enzymes that cleave both strands of a DNA double helix, pass another duplex through this transient gate and reseal the break. Type-2 topoisomerases are the enzymes responsible for unknotting DNA, and, in *E. coli*, the responsibility falls solely on topoisomerase IV [21]. The loss of topoisomerase IV activity has additional affects in cells that include hyper-negative supercoiling and the inability to segregate newly replicated DNA [22-24]. Therefore, the effects of knots needed to be evaluated separately from supercoils and catenanes.

Here we use the previously characterized Hin site-specific recombination and DNA knotting system [21,25,26] to understand how the physical constraints placed upon intracellular DNA can alter its activity. This system ties knots topologically identical to those observed *in vivo *[6,8,10]. Although studying the effects of knots in chromosomal DNA would be optimal, it is not technically feasible because there is no direct way to measure chromosomal knotting. Therefore, we have examined what happens when DNA strands collide to recombine and knot a 5.4 kb plasmid containing a gene required for cell survival. Plasmids appear to be a reasonable model for chromosomal metabolism. For example, supercoiling changes in reporter plasmids [24] mirror changes in the supercoiling of the chromosome [27,28]. The recombined plasmid products generated by Hin are easily analyzed because of their small size. A recombination event occurring in the chromosome would be much more difficult to detect. Although Hin recombines and knots at the *hix *sites, the resulting knots can move during DNA metabolism. On the chromosome, this knot sliding could be as far as the size of a topological domain, ~10 kb [29], which would be more difficult to detect experimentally.

Here we show that Hin-mediated site-specific recombination and knotting led to dysfunction of the replicon and blocked expression of a gene on the plasmid. This process is highly mutagenic, and our results suggest that unless recombination and knotting are carefully controlled, intracellular DNA can be unstable. We suggest that such instability of the genetic material could help drive evolutionary variation.

## Results

### Experimental strategy

The experimental approach we use here to study the cellular effect of recombining and knotting DNA is outlined in Figure 1A. We have shown previously that Hin recombines and knots plasmid DNA in *E. coli *that topoisomerase IV unties [21]. The Hin site-specific recombination system models two *in vivo *processes: it tangles the DNA to create knots identical to those formed inside the cell and shuffles the DNA sequence to model DNA recombination [6-10]. The *hin *recombinase gene is provided by the plasmid pKH66 (hereafter referred to as pHIN) and is expressed from the *tac *promoter following induction by isopropyl-β-D-thiogalactopyranoside (IPTG). pHIN also encodes for spectinomycin resistance. *E. coli *cells harboring pHIN also contained either pBR322 (pBR), which lacks recombination sites and serves as a negative control, or one of two pBR22-derived plasmids pTGSE4 (pREC) or pRJ862 (pKNOT) that carry sites recognized by the Hin recombinase. All three plasmids contain the *bla *gene, which encodes β-lactamase and provides resistance to ampicillin. We used the *bla *gene as a reporter to assess the effects of recombining and knotting the DNA.

Hin binds two 26-bp recognition sites and makes double-stranded breaks at the center of these sites leaving a two-bp overhang within each break [30,31]. Next, Hin rotates the DNA strands in a right-hand direction as dictated by the required DNA negative supercoiling [26]. If both sites are wild-type *hix *(or *gix*) sites (black arrows Figure 1A), as in pREC, the two-bp overhangs are complimentary and religation may occur after a 180° rotation. However, if the Hin substrate has one wild-type and one mutant site (grey arrow Figure 1A), as in pKNOT, the overhangs are not complimentary and a 360° rotation (or some multiple of 360°) is necessary for religation to occur. Thus, Hin recombines pREC and knots pKNOT. Although we initially anticipated that pREC would serve to differentiate between effects caused by recombination and those caused by DNA knotting, we (data not shown) and others observed that, *in vivo*, Hin will occasionally mediate processive recombination events to knot plasmids containing wild-type recombination sites at a steady-state level of 2 – 3% (Table 1) [25]. pKNOT is extensively knotted by Hin because the mismatch between the sites requires processive recombination [21,25,26] for religation. Hin expression increases the steady-state knotting of pKNOT approximately 5- to 10-fold over endogenous levels in the presence of topoisomerase IV function and 35- to 45-fold when topoisomerase IV function is blocked [21,25]. Twist knots with three (3_1_), five (5_2_) and seven (7_2_) negative nodes as well as composite knots with six (3_1_#3_1_), eight (3_1_#5_2_) and nine (3_1_#3_1_#3_1_) nodes are readily generated when Hin knots pKNOT [25]. Higher noded knots are seen in decreasing abundance. Only twist knots with three (3_1_) and four (4_1_) nodes are seen when Hin knots a plasmid with two wild-type recognition sites [25]. Previous studies examined the effect of Hin and other site-specific recombinases on gene expression [32,33]. However, a key distinction between those studies and the experiments performed here is that in those experiments the recombinase binding sites were placed between the promoter and the gene whereas here the reporter gene is distant to the site of recombination. Thus, here we examine the global effects on the DNA molecule rather than the local effects on promoter function.

**Table 1:** Hin-mediated knotting.

	···-*bla*-···	···-*bla*-AA–TT-···	···-*bla*-AA–AT-···
*in vivo*	< 1% [6]^a^	N.D.	5–10% [21]
*in vivo *+ NOR^b^	N.D.	2–3% [25]	35% [21], 45% [25]
*in vitro*	N.D.	5–10% [26]	> 80% [26]

^a^no Hin induction

^b^norfloxacin (to block unknotting by topoisomerase IV)

### Hin-mediated recombination and knotting of a plasmid alter function of a reporter gene

We first assessed the effect of Hin-mediated DNA recombination and knotting on resistance to ampicillin conferred by the *bla *gene on pBR, pREC and pKNOT. LB-agar contained a gradient of ampicillin [34], a constant concentration of spectinomycin to maintain the Hin expression vector and IPTG to induce expression of Hin. Wild-type *E. coli *K12 strains, C600 or W3110, containing pHIN and either pBR, pREC or pKNOT were streaked across the LB-agar. Whereas the strains containing pBR and pREC were able to grow on the highest ampicillin concentrations, growth of the strain carrying pKNOT was limited (Figure 1B). We determined whether this effect was specific to the gene encoded by the plasmid being targeted, pKNOT, or caused a general increased susceptibility to antimicrobial agents. Knotting (and recombination) had no effect on resistance encoded on a separate plasmid or on the chromosome: strains harboring the three plasmids were all growth inhibited at identical concentrations of spectinomycin (resistance encoded by pHIN) or norfloxacin (targets encoded by the chromosome) (data not shown). These results indicate that the sensitivity of *E. coli *to ampicillin is affected negatively when a knotted plasmid encodes its resistance. Knots impair the function of the replicon on which they form rather than cause a general effect on the cell.

We determined minimal inhibitory concentration (MIC_50_) values (the ampicillin concentration that inhibits 50% of bacterial growth) to quantify the Hin-mediated sensitivity to ampicillin. Strains harboring pKNOT were killed at a lower ampicillin concentration (1.4 mg/ml) than pBR (4.7 mg/ml) or pREC (2.3 mg/ml) (Figure 1C). The intermediate sensitivity of the pREC-containing strain to ampicillin compared to the pBR- and pKNOT-containing strains could be caused either by the intermediate level of DNA knotting that occurs in pREC or by Hin-mediated recombination of pREC.

### Hin recombination and knotting alter β-lactamase levels

**Figure 2:**
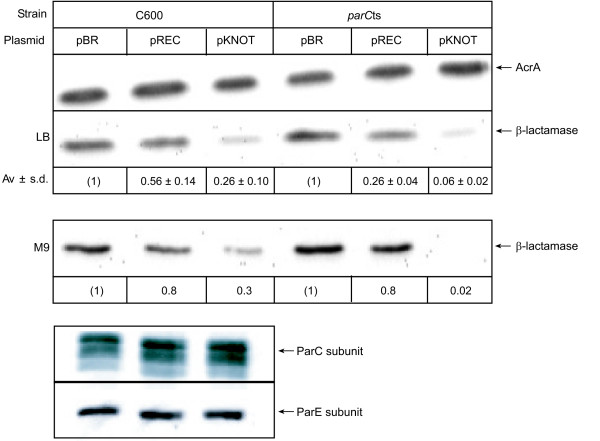
**Hin-mediated effect on β-lactamase protein levels**. Cultures of C600 (left) and *parC*ts (right) were grown in rich (LB) or minimal (M9) medium. Results here are from a typical experiment performed at 42°C. Immunoblotting was performed on total cellular lysates. Blots were probed with anti-AcrA, anti-β-lactamase, anti-ParC or anti-ParE antibodies. Shown below the blot from cells grown in LB is the mean of four independent experiments (except for *parCts *pREC, which was performed three times) and standard deviations. The values below the M9 experiment show the quantification of that blot, but the M9 experiment was repeated with the same results.

To dissect the molecular mechanism by which Hin affects the function of the *bla *gene on pREC and pKNOT, we performed immunoblots to assay β-lactamase levels. Strains were grown in liquid medium containing IPTG and spectinomycin until mid-logarithmic phase (OD_600 _= 0.3). Equal amounts of whole cell extracts were submitted to SDS-PAGE and either stained with Coomassie blue or subjected to Western blotting with anti-β-lactamase antisera (Figure 2). The Coomassie blue stained gels indicated that equal amounts of protein had been loaded (data not shown). The pHIN-containing C600 strain with pKNOT produced four- and three-fold less β-lactamase than the pBR- or pREC-containing strain, respectively, in either rich (LB; Figure 2A) or minimal M9 medium (Figure 2B). The reduction in β-lactamase levels correlates well with the reduction in ampicillin resistance (compare Figure 1C with Figure 2). There was no effect of pKNOT on levels of the chromosomally encoded topoisomerase IV subunits, ParC or ParE, or on levels of AcrA (Figure 2 and data not shown). Therefore, the reduction in protein levels is specific to genes encoded on the plasmid rather than a general inhibition of gene expression. Subsequently, AcrA levels were used to standardize loading.

If it is knotting that caused the increased susceptibility to ampicillin, then, because topoisomerase IV resolves knots in *E. coli*, inhibiting topoisomerase IV should increase the amount of knots and cause an additional reduction of β-lactamase production from pKNOT. To test whether knotting increased ampicillin susceptibility, we utilized a temperature-sensitive topoisomerase IV mutant, *parC*ts. Although cell growth and viability are reduced at the non-permissive temperatures for *parC*ts, cell division continues to occur and produces enough viable offspring that we were able to obtain sufficient growth (OD_600 _= 0.3) to perform immunoblots. When either of the non-permissive temperatures, 37°C or 42°C, for the *parC*ts allele was used, the results were the same. pKNOT in the *parC*ts strain produced 8.5-fold less β-lactamase than pREC and 17-fold less than pBR when cells were grown in LB or M9 medium at the non-permissive temperature (Figure 2, right panels). Therefore, inhibiting the enzyme that unties the knots exacerbates the reduction in β-lactamase production. As with the MIC_50 _data above, it is unclear whether the β-lactamase differences between pBR and pREC are caused by Hin binding, recombining or knotting pREC. There is less β-lactamase produced in the *parC*ts harboring pKNOT than C600 containing pKNOT. However, the plasmids carried in the two strains have different superhelical densities. In the *parC*ts strain, DNA is more negatively supercoiled at the non-permissive temperature [24]. The increased negative supercoiling should, if anything, slightly stimulate β-lactamase production. However, the knots counter this increase in β-lactamase production. Thus, the inhibitory effects of DNA knotting may be greater than measured because some effects are potentially being masked by the increase in negative supercoiling.

### Molecular analysis of Hin-mediated effects

It has been observed *in vitro *that DNA knots can diminish transcription [35]. Thus, the effect on β-lactamase production and ampicillin resistance we observed could be explained by an inhibition of *bla *transcription. However, it could also result from knots interfering with DNA replication, which would reduce the number of copies of the *bla *gene and, consequently, the amount of β-lactamase generated. An effect of DNA knotting on replication *in vitro *or *in vivo *has not been documented previously. Additionally, knots could reduce *bla *expression by causing pKNOT to break by weakening the tensile strength of DNA. Precedence of knots weakening and breaking polymers has been predicted by molecular dynamics simulations of polyethylene chains [36], shown using optical tweezers on actin filaments [37] and demonstrated with soft macroscopic strings [38] and with fishing line [39]. It is possible that Hin mediates its effects through a combination of blocking transcription, interfering with replication and breaking pKNOT.

**Figure 3:**
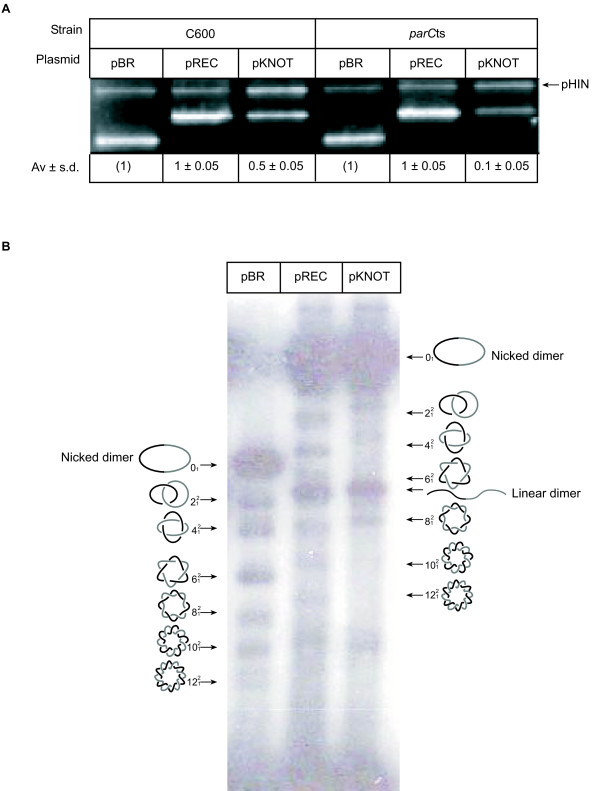
**Hin-mediated effect on plasmid replication**. (A) Plasmid DNA was isolated from strains C600 or *parC*ts containing the plasmids indicated. The DNA was linearized with *Hind*III, which cuts all the plasmids once, including pHIN. Shown is a representative ethidium bromide-stained agarose gel from an experiment performed at 42°C. Given below the gel image are the mean plasmid level values of three independent experiments with standard deviations. (B) Plasmid DNA was isolated 50 min. after IPTG induction of Hin, nicked, displayed by high-resolution gel electrophoresis and visualized by Southern blotting. Shown is an autoradiogram. All lanes contain plasmid DNA from the pHIN-harboring *parC*ts cells with pBR, pREC or pKNOT. The positions of nicked dimer (), linear dimer () catenanes (*e.g*., ) are indicated.

To determine whether plasmid stability and copy number are affected by Hin activity we quantified the levels of pBR, pREC or pKNOT DNA. Cultures were grown to mid-logarithmic phase and divided in half. Plasmid DNA levels were measured from one half and β-lactamase levels from the other half. DNA levels were determined by densitometric analysis of agarose gels. Following Hin induction, cells contained roughly two-fold less pKNOT than pBR or pREC relative to pHIN (Figure 3A). There was even less pKNOT DNA isolated from the *parC*ts strain grown at 42°C (Figure 3A, right side). When knotted, the copy number of pKNOT was reduced from ~20 – 40 copies/cell [40,41] to lower than pHIN levels in *parC*ts cells (Figure 3A). β-lactamase levels were determined from immunoblotting and densitometry of total cell lysates following SDS-PAGE. Although the DNA knots caused a reproducible reduction of pKNOT copy number, the magnitude of this effect (two- to six-fold) was never as large as the effect on β-lactamase levels (eight- to twenty-fold). In addition, pREC copy number was unchanged although there was a less than two-fold decrease in β-lactamase production from pREC in *parC*ts cells at 42°C. Therefore, decreased β-lactamase levels seen for pREC must not be at the level of replicon copy number. The difference between the effect on β-lactamase levels and the effect on plasmid copy number suggests that DNA knots mediate their effects through a combination of promoting replicon loss and blocking gene transcription.

It was possible that some cells had lost all their plasmid DNA to become plasmid free and other cells retained normal plasmid levels or that all cells generally had reduced plasmid levels. To distinguish between these possibilities, we grew cells harboring pHIN and either pBR, pREC or pKNOT in the presence of IPTG and spectinomycin, under conditions identical to those used to evaluate plasmid levels and β-lactamase production, except ampicillin was not included. Cell culture dilutions were spread on LB-agar and incubated overnight at 30°C. The following day, the colonies were replica plated onto LB-agar ± ampicillin, grown overnight at 30°C and counted. The frequency of plasmid-free cells was the same among all the three strains and was similar to what others have observed for loss of pBR in *E. coli *grown in a rich medium (LB) over the time period comparable to the one used here (~3 h) [42]. Thus, in these experiments, the Hin-mediated effect does not lead to complete loss of pKNOT. However, it is possible that given enough time complete plasmid loss might occur.

DNA catenanes are produced as intermediates of replication and they accumulate in temperature-sensitive topoisomerase IV mutants at the non-permissive temperature [22,23]. When DNA replication is disrupted, replication catenanes do not accumulate ([22] and data not shown). We examined the levels of catenanes in *parC*ts carrying pHIN and either pBR, pREC or pKNOT. Plasmid DNA was isolated from *parC*ts strains grown for 40 min. at 42°C as before, nicked to remove supercoiling and analyzed by high-resolution agarose gel electrophoresis [43]. Catenated pBR and pREC products were clearly visible, but DNA catenanes were greatly reduced for pKNOT (Figure 3B). Because catenanes were seen under conditions where either *bla *was fully functioning (pREC) or impaired (pKNOT), it does not seem likely that catenanes block replication and transcription (or cause mutagenesis). This experiment also provided an indirect method to examine the effect of knotted DNA on DNA replication. Because DNA replication is the only source of the catenanes, the observation that the level of DNA catenanes is reduced provides additional support that DNA replication is impaired in pKNOT.

### Hin-mediated recombination/knotting is mutagenic

**Figure 4:**
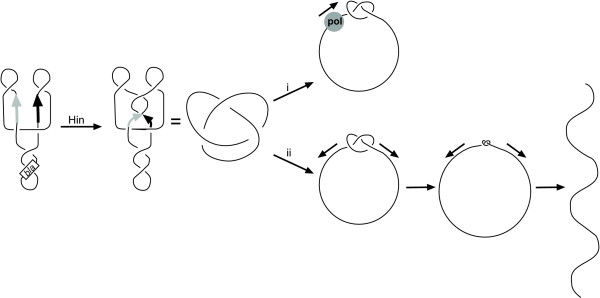
**Potential models for the Hin-mediated effect**. Plasmid pKNOT is recombined by Hin to knot the DNA (a single line represents the central axis of the double helix). In the roadblock model, the knot (or possibly Hin bound to or cleaving DNA) is impassable and stalls polymerase. Alternatively, in the breakage model, knots may break DNA as a result of forces on the plasmid.

We propose two models to explain how Hin blocks the function of the *bla *gene (Figure 4). These possibilities are not mutually exclusive. The first is the "roadblock" model: Hin, bound to or cleaving DNA, or the knots themselves form an impasse to RNA and/or DNA polymerases. The second is the "breakage" model. Although it is not clear how knots localize in DNA, it has been suggested from numerical studies that knots may spontaneously pull into a tight conformation [44,45]. DNA within the cell is constantly being subjected to a number of pulling forces generated by transcription, replication and segregation. A force (15 pN) comparable to that generated by a single replication or transcription complex [46-49], has been shown to tighten a DNA trefoil to a diameter less than 25 nm [37,50]. Either the roadblock or the breakage model predicts that DNA knots would be mutagenic through replication fork arrest or through a DNA double-strand break. In addition, either could induce the SOS response, which could lead to a genome-wide increase in mutation frequency [51,52].

**Figure 5:**
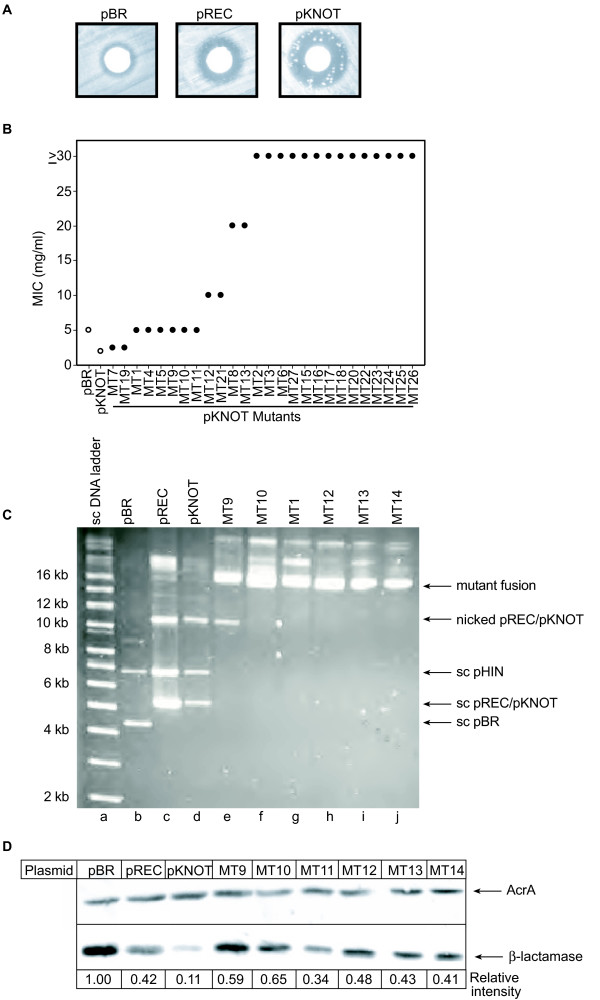
Hin-mediated mutagenesis. (A) Ampicillin resistant colonies growing in the zone of clearance around a filter containing 4 mg ampicillin. (B) Quantitation of ampicillin resistance of individual colonies. (C) Ethidium bromide-stained gel of plasmid DNA isolated from mutant colonies growing within the zone of clearance and separated by agarose gel electrophoresis. Lane a is a supercoiled molecular weight standard. Lanes b, c and d contain plasmid DNA from the parental strains harboring pHIN and either pBR, pREC or pKNOT, respectively. Lanes e-j contain plasmid DNA isolated from mutant pKNOT colonies. (D) Total cell lysates of mutants grown in 1 mM IPTG were separated by SDS-PAGE and submitted to immunoblotting. Immunoblots were probed with anti-AcrA antibodies (for a loading control) or anti-β-lactamase antibodies. Shown below the blot are signal intensities in arbitrary units. AcrA and anti-β-lactamase levels for C600 strains containing pHIN and either pBR, pREC or pKNOT are shown for comparison.

While measuring the ampicillin resistance of pBR-, pREC- and pKNOT-containing cells, we observed that, following overnight growth, C600 or *parC*ts strains harboring pKNOT formed large, robust colonies in the zone of drug-mediated clearing (Figure 5A). In contrast, no colonies were observed in the cleared zones around filter discs containing any concentration of ampicillin in lawns of C600 or *parC*ts strains containing pBR or pREC (Figure 5A and data not shown). We found that the effect was specific to β-lactam (ampicillin or cefotaxime) resistance, as no colonies were found in the zones of drug clearance for pBR-, pREC- or pKNOT-containing C600 or *parC*ts when norfloxacin was used (data not shown). These results suggest that increased mutation rate is occurring specifically for pKNOT and not the genome as a whole.

One hundred percent of the colonies that grew in the drug-cleared zone had a higher resistance to ampicillin than the original pKNOT-containing C600 cells and 69% had higher resistance than pBR-containing cells (Figure 5B). Using the MIC_50 _values of the original pBR, pREC and pKNOT strains (Figure 1C), we performed fluctuation assays to determine the mutation rate to ampicillin resistance (Table 2). Ampicillin at 4.8 mg/ml (3.4-fold higher than the MIC_50 _for pKNOT) was high enough to block all growth and select for hyper-resistant mutants in the C600 strain containing pHIN and pKNOT. At this concentration of ampicillin, using the MSS maximum likelihood method [53], the pKNOT strain yields 3.4 × 10^-6 ^mutations/cell/generation. At an ampicillin concentration of 16.1 mg/ml (3.4-fold higher than the MIC_50_), C600 containing pHIN and pBR yields 4.8 × 10^-10 ^mutations/cell/generation. At an ampicillin concentration of 7.9 mg/ml (3.4-fold higher than the MIC_50_), C600 containing pHIN and pREC had a mutation rate of 4.7 × 10^-7 ^mutations/cell/generation. Thus, Hin-mediated recombination and knotting increased the mutation rate three to four orders of magnitude compared to the spontaneous mutation rate of cells with pBR and Hin expression (Table 2).

**Table 2:** Hin-mediated mutation rates

**Selective AMP concentration, mg/ml**	**Plasmid**	**Resistant bacteria**		**Mutation rate per cell division**	**Mutation rate normalized to pBR**
				
		**Zero Fraction**	**Median**		
16.1	pBR	8/10	625.7	4.8 × 10^-10^	1
7.9	pREC	0/10	4817.1	4.7 × 10^-7^	969
4.8	pKNOT	0/10	6852.0	3.4 × 10^-6^	7106

To determine the molecular basis for the hyper-resistance to ampicillin, plasmid DNA was isolated from mutant colonies and analyzed (Figure 5C). There were two notable and unanticipated features of these rearrangements. First, the isolated plasmid DNA was much larger than the parental pKNOT. This result was surprising because any number of deletions or substitution mutations could disrupt Hin recombination and these types of changes would either result in a smaller plasmid or no change in plasmid size. However, these latter types of alterations were not apparent. Second, we found that not only was pKNOT altered, but pHIN was also changed in the hyper-resistant mutants. Gross genetic rearrangements of the plasmid were visible by restriction endonuclease digestion of each sample (data not shown). These results suggest that recombination between pHIN and pKNOT is responsible for the rearrangements and the phenotype of hyper-resistance to ampicillin. Although Hin does not directly recombine or knot pHIN, it is likely that recombination between pHIN and pKNOT results in a fused plasmid that is either refractory to additional knotting/recombination or expresses β-lactamase at a sufficient level to confer hyper-resistance to ampicillin. Without causing ampicillin hyper-resistance, pKNOT-pKNOT fusions would not be selected. In an attempt to analyze the role of homologous recombination in this plasmid rearrangement, we tried, but were unable to transform mutant strains lacking *recA *or *recD *with pHIN.

The plasmid changes and ampicillin hyper-resistance were heritable. Plasmid DNA was isolated from the colonies that arose in the zones of clearance and transformed into C600 cells harboring pHIN. The plasmids conferred a higher level of ampicillin resistance than pKNOT as determined by Kirby-Bauer assay (data not shown). We found that in four of five transformants tested, the mutant plasmid-transformed cells retained hyper-resistance to ampicillin and there were no visible colonies in the new zones of clearance. Because of this, it appears that either no further DNA rearrangements are occurring or, if they are, these additional rearrangements do not confer ampicillin hyper-resistance. The transformant (1/5) that behaved similarly to pKNOT-containing strains indeed harbored pKNOT. Thus, the fused mutant plasmid appears to have resolved back into pHIN and pKNOT. To compensate for reduced production of β-lactamase, the mutant plasmids could contain either a mutated *bla *gene that produces an enzyme more efficient at metabolizing ampicillin, or the mutations could allow for increased production of the enzyme. Using immunoblot analysis as described above, we found that all the cells carrying the rearranged plasmids that were examined had increased β-lactamase production relative to pKNOT (Figure 5D).

To determine whether the larger molecular weight plasmid that had replaced pKNOT and pHIN contained DNA originally present in both pHIN and pKNOT, we transformed C600 with total plasmid DNA from the ampicillin hyper-resistant isolates. Plasmid DNA from four of the ampicillin resistant colonies was used in independent transformations. For each transformation, half of the transformed cells was spread on LB-agar containing ampicillin (100 μg/ml, sufficient to select for the parental pKNOT), and half was spread on LB-agar containing spectinomycin (50 μg/ml, to select for pHIN). We found that 64/64 spectinomycin resistant transformants were also resistant to ampicillin and 28/32 ampicillin resistant transformants were also resistant to spectinomycin. These results are consistent with a fusion between pHIN and pKNOT being responsible for the ampicillin hyper-resistance phenotype observed in the majority of mutants.

## Discussion

Intracellular DNA is supercoiled, compacted and highly concentrated. Consequently, DNA will collide frequently with itself, and the result of these collisions increases the potential for DNA recombining and knotting. We have analyzed what can happen when the collisions lead to recombining and knotting. The results are that both replication and transcription are blocked and genetic rearrangements are increased.

### Mechanism of the Hin-mediated effect

DNA knots, and not recombination, are likely the predominant cause of Hin-dependent replication and transcription blocks and mutagenesis because the effect for pKNOT is more severe than for pREC. The effects were not caused by inherent differences in the three plasmids. Ampicillin MIC_50 _values of C600 strains harboring pHIN and either pBR, pREC or pKNOT grown in the absence of IPTG were identical (data not shown). In addition, the magnitude of the pKNOT-mediated effects was increased by compromising the activity of the enzyme, topoisomerase IV, responsible for unknotting DNA. However, in addition to unknotting, topoisomerase IV carries out two other cellular tasks: decatenation (reviewed in [54]) and DNA supercoil relaxation [24,55]. Removal of the decatenation activity of topoisomerase IV did not account for the increased pKNOT-mediated effects because far more catenated replication intermediates were seen in *parC*ts cells containing either pBR or pREC than in those that contain pKNOT (Figure 3B). The DNA supercoiling shift resulting from the inhibition of topoisomerase IV is not enough to stimulate either the transcription of the supercoiling-dependent *leu-500 *promoter [24] or the λ integrase recombination system [56]*in vivo*, suggesting that the increase in negative supercoiling resulting from inhibiting topoisomerase IV activity is unlikely to affect Hin recombination. It is possible that mechanistic differences in recombination on a substrate with two wild-type sites (pREC) compared to a substrate with one wild-type and one mutant site (pKNOT) could account for the Hin-mediated effects. For example, in a purified system, DNA cleavage by Hin is stimulated by a single mutant recombination site [31]. Additionally, *in vivo*, DNA cleavage of pKNOT by pHIN has been detected [25].

Plasmids replicate completely in less than six seconds and do so asynchronously. Moreover, they transcribe constantly. Thus, a slight increase of a lethal DNA form could have large consequences. Although topoisomerase IV rapidly unties knots, perhaps knot-induced problems, such as stalled replication forks, or stalled or blocked transcription, persist longer than the knots themselves. Indeed because topoisomerase IV can resolve DNA knots as they are formed, then, as the copy number of the plasmid goes down, there should come a point at which topoisomerase IV can resolve all the knots produced by the Hin system. The result would not be a complete loss of plasmid, but instead a steady-state level lower than that found with unknotted DNA, which is what we observed (Figure 3A). It is difficult to envision a process analogous to topoisomerase IV unknotting that would reverse the effects of Hin-mediated site-specific recombination. Thus, if recombination were leading to the loss of plasmids, it would seem that the unchecked altered plasmid would be lost completely from a population of cells in the absence of selection, which was not observed.

The DNA knot- or recombination-created blockage could impinge upon either the initiation or elongation of gene transcription or DNA replication. Gene promoters and replication origins are small relative to plasmids. Unless DNA knots preferentially form in or are localized to promoters or origins, or are hotspots for recombination, then it is expected that the polymerase roadblocks would occur at arbitrary positions on the DNA. Thus, such blockages would likely be outside of where transcription or DNA replication initiates.

It has been demonstrated that when topoisomerase IV activity is reduced by mutation, *priA*, which encodes the PriA protein that plays an important role in restarting blocked replication forks, becomes an essential gene [57,58]. It is possible that the stalling of replication forks at knots is the cause of this need for PriA and would explain why the presence of gyrase, which can remove positive supercoils, but not knots, is insufficient to keep replication moving in these cells.

### Implications for cellular physiology and evolution

Given (i) the abundance of recombinases, transposases and topoisomerases found in both prokaryotic and eukaryotic organisms, (ii) the lack of sequence specificity by these enzymes, (iii) the confined space for the chromosomes and (iv) the propensity of DNA to react and entangle with itself, DNA rearrangements that lead to cellular transformation or death, or that contribute to the mutations that shape evolution seem likely to occur. In other words, an intrinsic lack of DNA stability might have helped drive selection and genetic change. In addition, cellular stress causes a number of recombinases and transposases to be activated [59,60]. Perhaps this activation creates a transient "hypermutable state" that allows cells to develop a mechanism to overcome the stress. Such an event would be similar to that suggested to occur during adaptive mutagenesis when *E. coli *are starved for lactose [61-63]. Consistent with this idea, cells harboring transposons such as Tn*10*, which can recombine and knot DNA, will out-compete cells lacking Tn*10 *that are otherwise isogenic, which suggests that the transposon confers a greater evolutionary fitness [64,65].

## Conclusion

Our results suggest that recombined and knotted forms of DNA are problematic for the cell. Thus, it is the DNA conformation, rather than the primary sequence, that causes malfunctions. Effects of transient changes in conformation may then persist through induced mutations in the primary sequence. Unexpectedly, the DNA molecule undergoing site-specific recombination/knotting can "attack" a bystander DNA, and thus both DNA molecules may be altered.

## Methods

### Strains and Plasmids

*E. coli *strains C600, ParC1215 (*parC*ts) and W3110 were described previously [21,66]. Plasmid pKH66 (pHIN) contains the *S. typhimurium hin *gene under control of the *tac *promoter and expresses Hin upon addition of isopropyl-1-thio-β-galactoside (IPTG) [21,67]. pTGSE4 (pREC) [68] is a pBR322-derived plasmid containing the Gin recombination (*gix*) sites and enhancer from bacteriophage Mu. Gin, Hin and their respective recombination sites are interchangeable [69]. pRJ862 (pKNOT) contains *hix *recombination sites and the enhancer binding site for the Hin recombinase from *S. typhimurium *[26]. One *hix *site contains a single base pair change, which forces a second round of recombination to tie knots by preventing religation after only one round [26]. To create the strains used throughout this work, we used a CaCl_2 _method to transform wild-type *E. coli *with plasmid DNA (typically 100 ng).

### Antibiotic resistance measurements

Gradient plates [34] and Kirby-Bauer [70] disc diffusion assays were used to measure antibiotic resistance. Saturated overnight cultures containing the strains were diluted 30- to 100-fold in LB containing 1 mM IPTG and 50 μg/ml spectinomycin. The freshly diluted cultures were grown at 37°C until they reached OD_600 _= 0.3. For the Kirby-Bauer disc diffusion assays, cells were spread on LB-agar containing 1 mM IPTG and 50 μg/ml spectinomycin. The plates were allowed to dry for 20 min. and discs containing 10 μl of different ampicillin concentrations (0 – 500 mg/ml) were placed onto the agar. The plates were then incubated overnight at 37°C. The diameter of the cleared zone around each disc was measured. For the gradient plate assay, cells were spread on square plates containing a gradient from 0 to 17.5 mg/ml ampicillin, and then incubated overnight at 37°C. The plate dilution method was used to determine the ampicillin MIC_50 _values. The three *E. coli *strains harboring pHIN and either pBR, pREC or pKNOT were grown overnight in LB medium containing 100 μg/ml ampicillin, 50 μg/ml spectinomycin and no IPTG. These cultures were diluted 500-fold in LB medium containing 50 μg/ml spectinomycin and 1 mM IPTG, but no ampicillin. The freshly diluted cultures were grown with shaking to mid-logarithmic phase (OD_600 _= 0.3 – 0.4) at 37°C. Appropriate dilutions (to final cell counts of approximately 100 and 1000 per plate) were spread onto LB-agar alone and LB-agar containing ampicillin concentrations from 1.3 to 4.8 mg/ml, 50 μg/ml spectinomycin, but no IPTG. Colonies were counted following overnight incubation at 37°C. For each of the three strains, regression analysis was performed to determine the best-fit curve through the data points (2670: n = 10, 2671: n = 9 and 2672: n = 8) in the plot of survival as a function of ampicillin concentration. From this best-fit curve, the ampicillin MIC_50 _values were extrapolated.

### Antibodies and immunoblotting

Isogenic C600 and ParC1215 (*parC*ts) strains were grown overnight in LB medium without IPTG. Cells were diluted 1/100 into LB medium and grown with shaking ± 1 mM IPTG and 50 μg/ml spectinomycin to mid-logarithmic phase (OD_600 _= 0.3 – 0.4) at 37°C or 42°C. Duplicate sets of whole cell extracts were made by resuspending equal amounts of pelleted cells in loading buffer (125 mM Tris-HCl, pH 6.8; 1.4 M β-mercaptoethanol; 20% glycerol; 2% SDS; 0.1% Bromophenol blue), boiling for 3 min. and subjecting to 10% SDS-PAGE. One set was stained with Coomassie blue to ensure equal protein amounts were loaded. The other set was blotted to a nitrocellulose Protran membrane. The blots were probed with (1:10,000 dilution for all) antisera to β-lactamase (a kind gift of T. Palzkill, Baylor College of Medicine, Houston), anti-AcrA (a kind gift of H. I. Zgurskaya, University of Oklahoma, Norman), anti-ParC or anti-ParE (kind gifts of the late N.R. Cozzarelli, University of California, Berkeley), incubated in SuperSignal West chemiluminescent reagent (Pierce, Rockford, IL) and visualized with a charge coupled display camera.

### Plasmid loss assay

Cells were grown as for Western blot analysis. Plasmid DNA was isolated by the alkaline lysis method [71], linearized with *Hind*III (which cuts pKNOT and pHIN once) and separated by electrophoresis on 1% agarose (TAE) gels. Plasmid levels were quantified by densitometric scanning (NucleoVision software, NucleoTech Corp., San Mateo, CA) of images of ethidium bromide-stained gels. Assuming pHIN levels do not change among the strains, plasmid bands were first normalized within each lane to the pHIN vector. These standardized band values are shown relative to the value for pBR within each strain background. To determine whether entire plasmid populations were lost from cells, various dilutions of cells grown in LB medium were spread onto LB-agar and replica plated on agar ± 100 μg/ml ampicillin. Colonies were counted following overnight incubation at 30°C (C600 and *parC*ts) or 37°C (C600).

### DNA catenane analysis

DNA catenanes were analyzed as done previously [22]. *parC*ts cells containing pHIN and either pBR, pREC or pKNOT were grown at 30°C to mid-logarithmic phase (OD_600 _= 0.3 – 0.4). IPTG was added to a final concentration of 1 mM to induce Hin expression. After 10 min., cells were shifted to 42°C to inactivate the mutant topoisomerase IV. Forty minutes later, plasmid DNA was isolated [71], nicked with DNase I to remove supercoiling [72] and displayed by high-resolution gel electrophoresis [22]. The DNA was then transferred to a Zeta Probe nylon membrane (Bio-Rad Laboratories, Hercules, CA) and probed with [α-^32^P]-dCTP (GE Healthcare, Little Chalfont, UK) labeled pBR322 (made by random priming, Amersham Megaprime™ DNA labeling systems, GE Healthcare, Little Chalfont, UK), which hybridizes all three plasmids.

### Isolation of ampicillin resistant colonies and fluctuation analysis

Ampicillin resistant colonies that grew inside the zone of clearance (Figure 5A) were streaked onto LB-agar plates containing 1 mM IPTG, 50 μg/ml spectinomycin and 1 mg/ml ampicillin and incubated overnight at 30°C. These conditions were used to prevent the accumulation of revertants to ampicillin sensitivity. To determine the mutation rate, *E. coli *harboring pHIN and either pBR, pREC or pKNOT were grown overnight in LB medium containing 100 μg/ml ampicillin, 50 μg/ml spectinomycin and no IPTG. The overnight cultures were diluted 6,000-fold into LB medium (~10^5 ^cells/ml) containing no ampicillin, 50 μg/ml spectinomycin and 1mM IPTG and divided into ten 1.2-ml aliquots. These aliquots were grown with shaking to mid-logarithmic phase (OD_600 _= 0.3 – 0.4) at 37°C to obtain parallel, independent cultures. The number of ampicillin resistant mutants that originated in each culture was determined by spreading 2 × 70 μl (pBR- and pREC-containing strains) or 2 × 200 μl (pKNOT-containing strain) of undiluted culture onto LB-agar containing various ampicillin concentrations, 50 μg/ml spectinomycin, but no IPTG. 16.1 mg/ml ampicillin was used for the strain harboring pBR; 7.9 mg/ml ampicillin was used for the strain harboring pREC; and 4.8 mg/ml ampicillin was used for the strain harboring pKNOT. Each of these ampicillin concentrations is 3.4-fold higher than the corresponding strain's ampicillin MIC_50_. The total number of cells was determined by spreading dilutions of each culture on LB-agar. Colonies were counted after incubation overnight at 37°C. The probable number of mutations per culture (m) was calculated from the distribution of hyper-resistant mutants in the independent cultures using the MSS maximum likelihood method. Then the mutation rate (μ) was calculated as μ = m/2N_t_, where N_t _is the total number of cells per culture [53].

## Authors' contributions

RWD carried out antibiotic resistance measurements, immunoblotting, plasmid loss assays, DNA catenane analysis and isolation of ampicillin resistant colonies, conceived of and participated in the design of the study and drafted the manuscript. JKM carried out antibiotic resistance measurements, plasmid loss assays, isolation of ampicillin resistant colonies and fluctuation analysis, performed the statistical analysis, participated in the design of the study and helped to draft the manuscript. DWS participated in the design and coordination of the study, analyzed experimental results and helped to draft the manuscript. LZ conceived of and participated in the design and coordination of the study, analyzed experimental results and helped to draft the manuscript. All authors read and approved the final manuscript.
